# The Influence of Whole Grain Products and Red Meat on Intestinal Microbiota Composition in Normal Weight Adults: A Randomized Crossover Intervention Trial

**DOI:** 10.1371/journal.pone.0109606

**Published:** 2014-10-09

**Authors:** Jana Foerster, Gertraud Maskarinec, Nicole Reichardt, Adrian Tett, Arjan Narbad, Michael Blaut, Heiner Boeing

**Affiliations:** 1 Department of Epidemiology, German Institute of Human Nutrition Potsdam-Rehbruecke, Nuthetal, Germany; 2 University of Hawaii Cancer Center, Honolulu, Hawaii, United States of America; 3 Gut Health and Food Safety, Institute of Food Research, Norwich Research Park, Colney, United Kingdom; 4 Department of Gastrointestinal Microbiology, German Institute of Human Nutrition, Potsdam-Rehbruecke, Nuthetal, Germany; The Chinese University of Hong Kong, Hong Kong

## Abstract

Intestinal microbiota is related to obesity and serum lipid levels, both risk factors for chronic diseases constituting a challenge for public health. We investigated how a diet rich in whole grain (WG) products and red meat (RM) influences microbiota. During a 10-week crossover intervention study, 20 healthy adults consumed two isocaloric diets, one rich in WG products and one high in RM. Repeatedly data on microbiota were assessed by *16S rRNA* based denaturing gradient gel electrophoresis (DGGE). A blood sample and anthropometric data were collected. Mixed models and logistic regression were used to investigate effects. Microbiota showed interindividual variability. However, dietary interventions modified microbiota appearance: 8 bands changed in at least 4 participants during the interventions. One of the bands appearing after WG and one increasing after RM remained significant in regression models and were identified as *Collinsella aerofaciens* and *Clostridium sp.* The WG intervention lowered obesity parameters, while the RM diet increased serum levels of uric acid and creatinine. The study showed that diet is a component of major relevance regarding its influence on intestinal microbiota and that WG has an important role for health. The results could guide investigations of diet and microbiota in observational prospective cohort studies.

**Trial registration:**

ClinicalTrials.gov NCT01449383

## Introduction

The composition of intestinal microbiota is gaining importance in human health as evidence is increasing that these bacteria play a role in disease aetiology [1]. Thus, it is important to increase knowledge in how lifestyle factors, e.g., diet, physical activity and smoking influence gut microbiota. This seems to be crucial since large observational studies, such as prospective cohort studies, aim at examination of the involvement of human microbiota in health and disease [2]. It is well established that the microbiome enables complex interactions between the 10^14^ cells of the intestinal microbiota and its host, including processes of fat storage, and maturation and maintenance of the immune system [3], [4].

One important factor determining microbiota composition from birth besides the host's genome is habitual diet since different foods primarily serve as substrate for microbial growth in the gastrointestinal system [5]. A major function of gut microorganisms is to digest food compounds that are not degraded by human enzymes. In this manner, compounds like complex polysaccharides and starch promote proliferation of certain bacterial populations, which in turn provide degradation products, e.g., monosaccharides or short chain fatty acids (SCFA) for subsequent absorption. Results of a former study in mice indicate that in this way a changed microbiota composition contributes to a higher energy yield, weight gain, and possibly obesity [6].

Prospective studies have revealed two food groups with opposing effects on diet-related diseases. Whole grain (WG) products (and the associated intake of dietary fibre) appear to reduce risk for metabolic disorders, whereas red meat (RM) is more likely to constitute a risk factor for chronic diseases [7]–[10]. Therefore, the present study investigates the effects of diets high in WG products and RM, respectively, on gut microbiota composition by an isocaloric intervention approach in healthy adults. The results will help to concentrate the statistical efforts of risk evaluation in prospective cohort studies on those groups of bacteria that are known to be directly modified by dietary changes.

## Methods

This crossover trial assessed the effects of WG and RM products on the composition of intestinal microbiota, anthropometry, several blood parameters and faecal biomarkers. The study protocol was approved by the ethics commission of the Brandenburg State Chamber of Physicians in October 2010 and the trial was conducted in accordance with the Declaration of Helsinki [11]. The study was conducted without deviation from the study protocol approved by the ethics commission. All participants signed an informed consent form. Because the trial was approved by an ethics commission already in October 2010 it was registered on ClinicalTrials.gov, NCT01449383, after starting the enrolment of participants in October 2011. The authors confirm that all ongoing and related trials for this intervention conducted by the department are registered. The protocol for this trial and the supporting CONSORT checklist are available as supporting information; see Checklist S1 and Protocol S1.

### 2.1 Study population and participant recruitment

**Figure 1:**
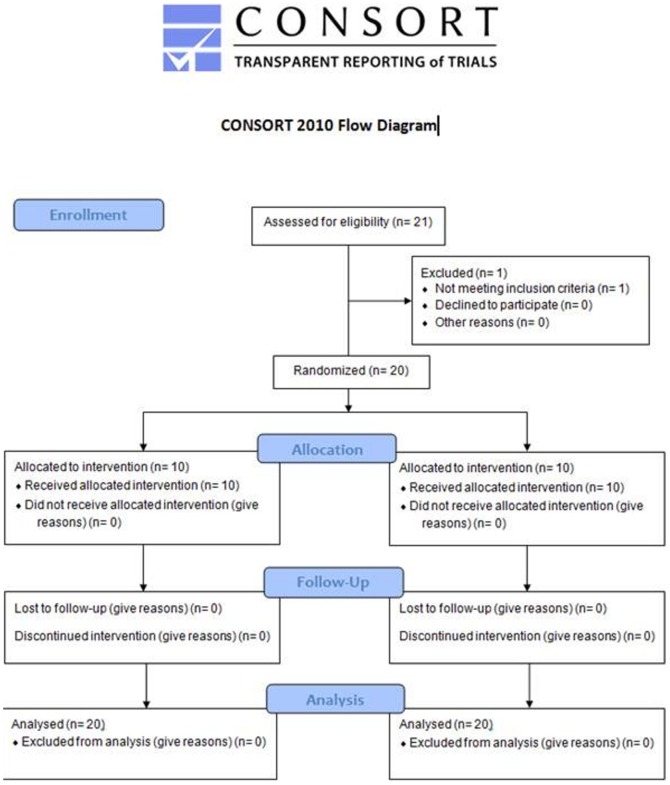
CONSORT flowchart of the study.

Between November 2010 and August 2011 twenty healthy free-living participants (Figure 1) with equal portion men and women aged 20 to 60 years were recruited by advertisement and flyers, which were spread in the public in the surrounding of the institute. Additionally invitations were sent via email. Exclusion criteria were acute or chronic gastrointestinal diseases, disorders, or surgeries, prevalent chronic diseases such as diabetes mellitus and cancer, antibiotic treatment during the last 3 month, pregnancy, and breastfeeding. All participants were asked to report the consumption of pre- and probiotic foods, such as yogurts, and supplements during the last 2 weeks before the intervention.

### 2.2 Study plan and interventions

**Figure 2:**
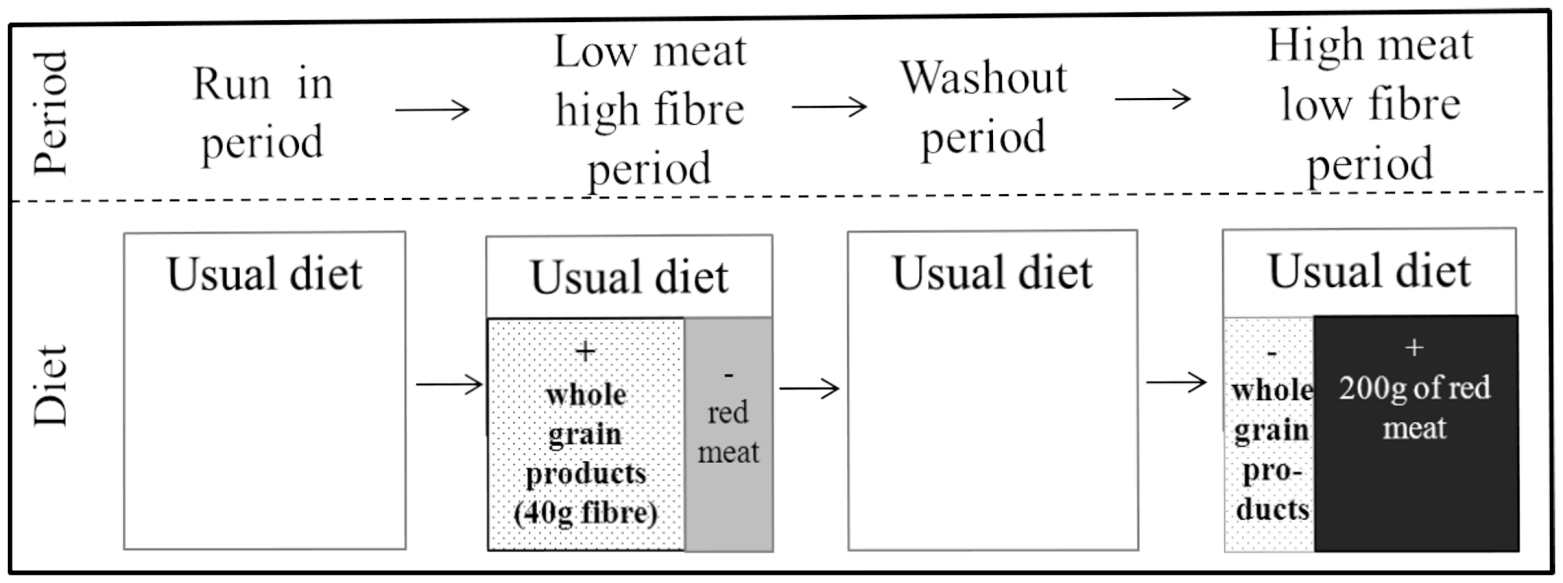
Diet plan for participants with diet succession: whole grain diet, washout period and red meat diet.

After recruitment and pre-study information sessions, the participants were instructed about their diet during the intervention. Successive order of invitation rendered an individual and detailed instruction possible. All participants obtained booklets containing information on the diet during the intervention periods. Every participant took part in both intervention periods lasting 3 weeks each seperated by a 3-week washout period (Figure 2). Sequence of diet was randomised via coincident allocation of the first diet and the two diets were isocaloric. The diet during the WG period required low intake of red meat products, i.e., not more than 30 g per day, and high amounts of WG products resulting in a daily intake of approximately 40 g dietary fibre (WG intervention). The other diet consisted of 200 g of RM per day and minimal amounts of dietary fibre (RM intervention) (Figure 2).

The participants were provided with the foods needed for the interventional diets free of charge. During WG, they received 3 types of bread rich in dietary fibre from the bakery ‘Maerkisch Landbrot’, (‘Bergroggenbrot’, ‘Roggensonne’ and ‘Korn&Kraeuterbrot’) with a fibre content between 12.2 and 15.2 g per 100 g. Additionally, a muesli from ‘Seitenbacher’ (Muesli 348 – Ballaststoffmischung, fibre content: 14.3 g/100 g) was supplied. For RM, portions of 200 g (fresh weight) of red meat, i.e., pork cutlet, beef steak and others were provided for every day of the intervention. In addition to receiving foods, participants were asked regularly about their consumption of the foods in order to assess adherence to the interventions. We decided against dietary assessment by questionnaire or recall due to the short intervention period and the highly conscientious participants. Instead, we relied on the detailed instructions, the food supplies and regular personal contact with the participants. Also we were only interested in the effects of red meat and whole grain consumption and not in the usual daily diet of the participants.

### 2.3 Data collection

Before and after each period participants have an appointment in the institute's study centre. At this body height, body weight, waist and hip circumference, and skin fold thickness on arm, back and hip were measured. Body mass index (BMI) [kg/m^2^], waist to hip ratio (WHR) and body fat mass [%] [12] were calculated. At the same time, a blood sample was drawn, which was analysed immediately. Additionally, all participants provided a faecal sample, which was collected at the participants' home, frozen immediately after collection at −20°C in a freezer, transported to the Institute without thawing within 3 hours, and stored at −80°C until analyses. Participants were examined between November 2010 and September 2011 according to the above mentioned timeframes.

### 2.4 Laboratory analysis

Blood samples were analysed for clinical blood parameters (C-reactive protein [CRP], total cholesterol, high density lipoprotein [HDL], low density lipoprotein [LDL], triglycerides [TG], uric acid and creatinine) using standard clinical procedures. The cholesterol quotient was calculated as the proportion of LDL to HDL.

The faecal microbiota composition was analysed by PCR-DGGE. Briefly, total DNA from faecal samples was extracted using the FastDNA Spin Kit for Soil with a modified protocol [13] and stored at −20°C until further analyses. The DNA was used as a template to amplify the variable regions V6-V8 of the bacterial *16S rRNA* genes with primers U968-GC-f (5′-CGCCCGGGGCGCGCCCCGGGCGGGGCGGGGGCACGGGGGGAACGCGAAGAACCTTAC-3′) and U1401-r (5′-CGGTGTGTACAAGACCC-3′) [14]. The G+C clamp at the 5′-end of the primer U968-GC-f is underlined. PCR amplification was performed as follows: initial denaturation at 94°C for 5 min, denaturation at 94°C for 30 s, followed by 35 cycles with an annealing temperature of 50°C for 20 s, and primer extension at 72°C for 40 s, and a final extension for 7 min at 72°C.

For DGGE analysis 150 ng of PCR product was analysed by using the DCode Universal Mutation Detection System as described previously with minor modifications. Briefly, a 40–60% gradient of urea and formamide was used and the gels were run in 1 x TAE buffer at 200 V for 10 min and then at 55 V for 16 h. DGGE gels were stained for 45 min in a Sybr Green solution (5 µl/300 ml H_2_O dest.), washed for 15 min in 300 ml H_2_O dest. and scanned with a Pharos FX scanner. DGGE gel analysis and inter-gel-comparisons were performed using the Phoretix 1D and 1D Pro software package as described by Tourlomousis et al. [15]. The analysis of the scans provided dichotomous information on the presence or absence of each potentially detectable DGGE band in each sample. Additionally, bands that were found to be changed after intervention periods were identified by sequencing the corresponding V6-V8 regions using primers U968 and U1401 and PCR conditions given above.

Further, faecal concentrations of SCFA (acetic, propionic, butyric, iso-valeric and valeric acid) were measured with gas chromatography with iso-butyric acid as internal standard [16]. Total SCFA concentration was calculated as the sum of all acids. Faecal calprotectin was measured with a commercially available monoclonal antibody-based enzyme-linked immunosorbent assay following the manufacturer's instructions. This analytic method has coefficients for intra-assay variation between 3.2 and 5.6% and for inter-assay variation between 4.4 and 8.9% [17]. All analyses were performed with thawed and homogenized faecal samples.

### 2.5 Statistical analysis

Potential differences in baseline characteristics between the two intervention groups were investigated with Student's t test for continuous variables and x^2^ tests for categorical variables. All variables were tested for normal distribution with the Shapiro-Wilk test; non-normally distributed variables (WHR, uric acid, creatinine, calprotectin, CRP, triglycerides, total SCFA and butyric acid) were log-transformed for further analyses. To assess intervention effects, mixed linear models were applied for continuous variables taking into account the dependence of observations within-person due to the repeated measurement design and least-square means by diet status (no intervention, RM, WG) were computed. For ‘no intervention’ status characteristics from baseline and washout period were combined if there exist no differences.

The descriptive analysis of gut microbiota included the relative abundance of bands and the diversity within samples expressed as total number of bands per sample. Since gut microbiota data were binary, the appearance of bands was evaluated as bands present before an intervention and absent afterwards (1→0) or vice versa (0→1). To identify bands that were influenced by one of the dietary interventions, we searched for bands, for which absolute changes into one direction were observed in at least 4 (20%) participants. This cut-off value was coincidentally the value for the corresponding McNemar test to be significant. Then, we carried out multivariate logistic regressions for all bands that fulfilled the above mentioned criteria with diet as the dependent variable.

As an exploratory analysis to evaluate correlations among data of DGGE bands that changed after an intervention, we calculated tetrachoric correlation coefficients considering corresponding DGGE bands and performed a factor analysis based on the resulting correlation matrix [18]. We used algorithms for unweighted least squares and the varimax method as an orthogonal rotation procedure considering that the inter-factor correlation was not higher than 0.13. Factors were extracted according to the criterion of maximal explained variance and corresponding factors were calculated for each sample. To evaluate whether all 80 observations of the 20 participants can be used to generate the tetrachoric correlations and the factors despite their dependent character intraclass correlation coefficients (ICC) were calculated. The influence of the dietary treatment on the final factors was investigated in mixed models. Finally, the relations between the factors of interest and several measures of obesity were assessed. In additional analyses relations between factors of interest and anthropometric parameters were adjusted for sex.

All statistical analyses were done in SAS Enterprise guide 4.3 and the level of statistical significance was p<0.05.

## Results

### 3.1 Baseline characteristics, anthropometric and blood parameters

Study participants had a mean age of 40 (11.6) years and a BMI of 24.4 (2.9) kg/m^2^ (Table 1). The two groups that started either with WG or with RM did not differ significantly in their baseline characteristics. Therefore, they were analysed together. Further, no significant differences between the investigated characteristics at baseline and after washout could be observed (p-values range from 0.09 to 0.86), therefore baseline and washout values were combined to ‘no intervention’.

**Table 1:** Baseline characteristics of study participants, overall and by intervention sequence group after randomisation; # Mean (SD), *pValues for ttest comparing continous variables in the groups and χ^2^test for categorical variables.

	Overall (n = 20)	WG → RM (n = 10)	RM → WG (n = 10)	*p* *
Age [years]^#^	40.1 (11.6)	37.8 (9.5)	42.3 (13.5)	0.4
Sex (male) [n(%)]	10 (50)	6 (60)	4 (40)	0.37
Microbiota diversity [total nb of bands]^#^	31.1 (5.8)	31.0 (4.1)	31.1 (7.4)	0.97
BMI [kg/m^2^]^#^	24.4 (2.9)	24.0 (2.5)	24.9 (3.4)	0.51
WHR^#^	0.86 (0.09)	0.85 (0.07)	0.87 (0.12)	0.66
Waist circumference category [n (%)]				
1 - ≤80/94 cm	13 (65)	6 (60)	7 (70)	
2 ->80/94 cm and ≤88/102 cm	5 (25)	3 (30)	2 (20)	
3 -> 88/102 cm	2 (10)	1 (10)	1 (10)	0.87
Body fat mass [%]^#^	28.7 (5.9)	30.0 (7.0)	27.4 (4.7)	0.34
Cholesterol [mmol/l]^#^	5.4 (0.8)	5.3 (0.8)	5.5 (0.9)	0.51
Quotient LDL/HDL^#^	2.4 (0.8)	2.3 (0.7)	2.6 (0.8)	0.33
Triglycerides [mmol/l]^#^	1.5 (0.8)	1.5 (0.8)	1.4 (0.7)	0.76
Uric acid [µmol/l]^#^	253.4 (81.0)	236.7 (69.6)	270.0 (91.6)	0.37
Creatinine [µmol/l]^#^	75.0 (14.4)	75.1 (16.4)	74.9 (12.9)	0.98
CRP [mg/l]^#^	2.1 (2.8)	2.3 (3.7)	2.0 (1.6)	0.76
Calprotectin [mg/kg]^#^	25.4 (21.5)	21.6 (26.3)	29.3 (15.8)	0.44
Total SCFA [mmol/g]^#^	37.0 (30.0)	47.1 (39.5)	26.9 (10.9)	0.14
Butyric acid [mmol/g]^#^	6.4 (6.3)	7.7 (8.5)	5.0 (2.8)	0.37

After the WG intervention, the BMI, body fat mass and body weight were statistically significant lower by 0.14 kg/m^2^, 0.77% and 0.45 kg, respectively (p = 0.05, p = 0.005 and p = 0.05, respectively), compared to values at baseline and after washout (Tab. 2). All other measured parameters remained unchanged. In contrast, no alterations in anthropometric parameters were observed after the RM intervention.

**Table 2:** Intervention effects on selected parameters from mixed effect models.

	No intervention*	Intervention effect			
		Red meat		Whole grains	
	Mean (SD)	Mean (SD)	*p* #	Mean (SD)	*p* #
Diversity [total nb of bands]	31.6 (5.5)	31.7 (5.6)	0.96	35.7 (6.6)	0.01
BMI [kg/m^2^]	24.5 (3.0)	24.4 (3.0)	0.64	24.3 (3.0)	0.05
Body fat mass [%]	28.6 (5.8)	28.6 (6.1)	0.91	27.9 (6.0)	0.005
Weight [kg]	75.4 (13.7)	75.3 (14.0)	0.66	74.9 (13.7)	0.05
WHR§	0.86 (0.09)	0.86 (0.09)	0.79	0.86 (0.09)	0.98
Uric acid [µmol/l]§	260 (79)	285 (83)	0.0002	261 (76)	0.68
Creatinine [µmol/l]§	74.4 (13.9)	79.4 (14.6)	0.0003	72.7 (12.2)	0.24
Calprotectin [mg/kg]§	41.2 (63.9)	29.0 (32.8)	0.09	32.6 (44.3)	0.77
CRP [mg/l]§	2.1 (2.4)	1.4 (1.2)	0.19	1.9 (3.8)	0.07
Cholesterol [mmol/l]	5.3 (0.8)	5.2 (0.8)	0.47	5.3 (1.0)	0.94
Quotient LDL/HDL	2.3 (0.7)	2.3 (0.6)	0.50	2.3 (0.8)	0.34
Triglycerides [mmol/l]§	1.4 (0.7)	1.5 (0.8)	0.68	1.6 (1.0)	0.11
SCFA [mmol/g dryweight]					
Total SCFA§	41.8 (34.9)	33.4 (20.2)	0.34	40.7 (25.5)	0.69
Butyric acid§	7.0 (6.0)	5.5 (4.6)	0.12	7.6 (5.8)	0.91

* Data from examination at baseline and after washout period combined.

# *p*-values less than 0.05 were considered significant.

§ *p*-values result from calculation of mixed models with log transformed variables.

The WG diet had no influence on blood levels of lipids and other blood parameters, but the RM intervention was associated with a significant increase in creatinine and uric acid of 6.8% and 10.0% (5.05 and 25.82 µmol/l; p = 0.0003 and p = 0.0002), respectively.

### 3.2 Intestinal microbiota and faecal parameters

**Figure 3:**
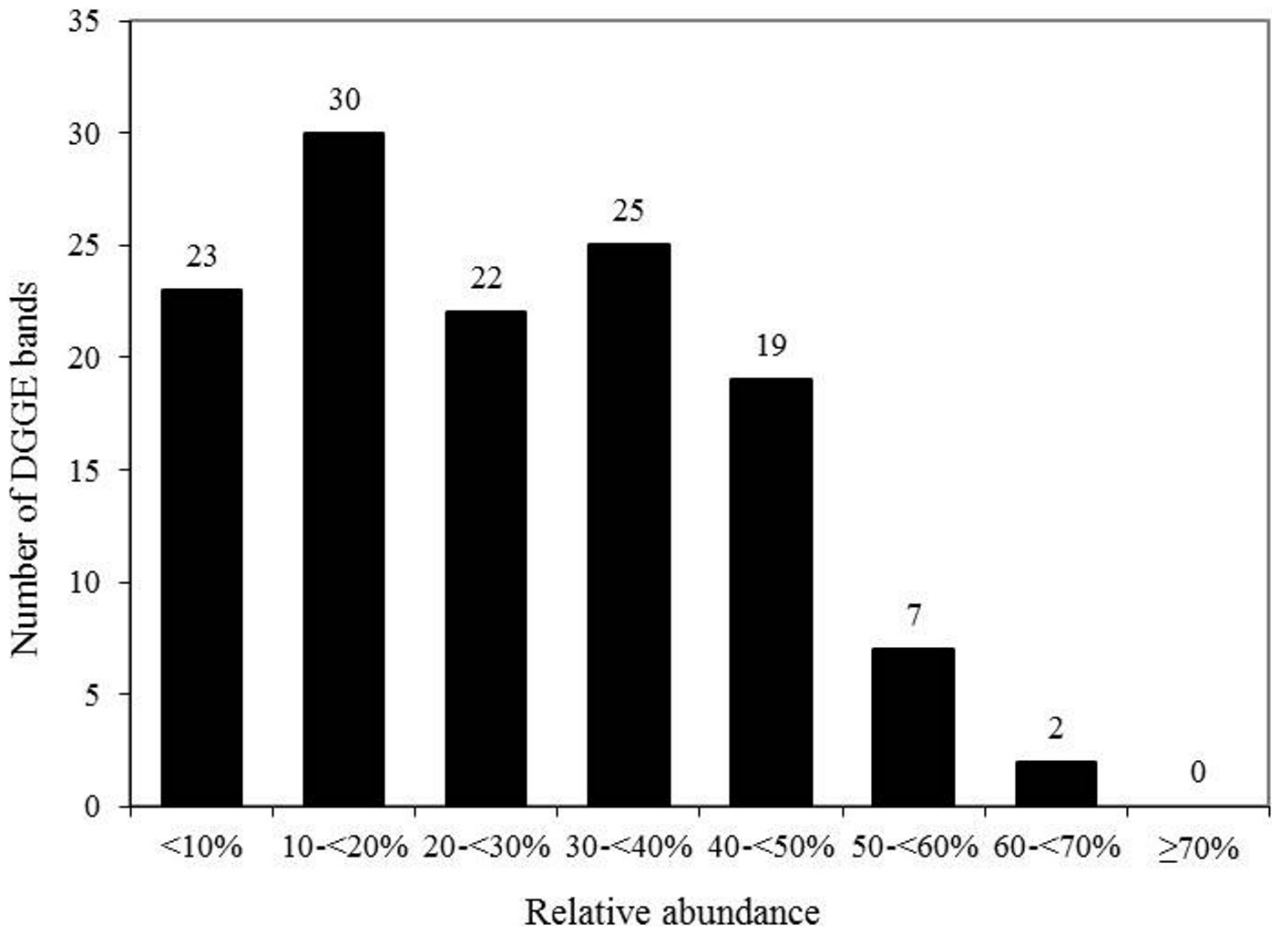
Relative abundance of DGGE-bands in human faecal samples from examinations before intervention periods.

Using DGGE, in total 128 different bands were detected. The microbial diversity ranged from 19 to 42 bands per participant at baseline with a mean of 31.1 (5.8) different bands (Table 1). A small proportion of 9 bands occurred frequently with a relative abundance of over 50%; most of the bands (96 bands) were less widespread and present in 10 to 50% of the participants (Figure 3).

**Figure 4:**
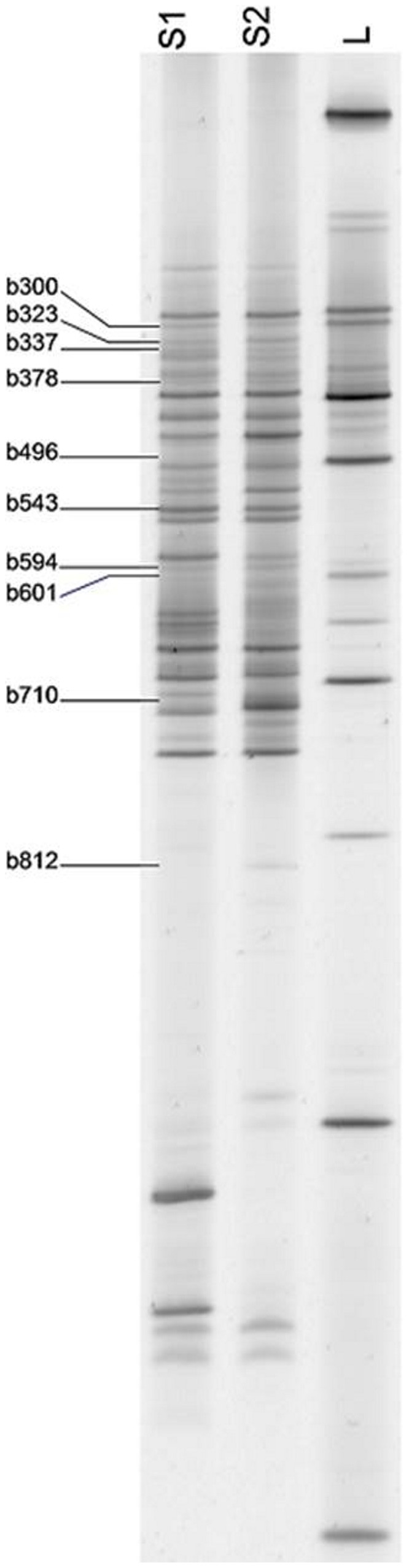
Position of DGGE bands with a significant change after intervention periods in a DGGE gel (L- Marker; S1/S2- Sample 1 and 2).

When bands were compared before and after the intervention periods, 8 bands changed in at least 4 participants during the RM as well as during the WG diet. Considering the high inter-individual variation this change was a significant difference (*p*Value of McNemat test ≤0.05) in consequence of the intervention. After the RM period, 5 bands increased and 3 bands decreased in their appearance, and the WG diet caused the reduction of 3 bands and the rise of 5 bands (Table 3, Fig. 4). The group specific analysis incorporating the intervention sequence showed that for all but 2 bands changes occurred in both groups (b300 and b935 only changed in the WG → RM group). The multivariate logistic regression revealed for each diet intervention only one band, b812 for WG and b496 for RM, which can be regarded as the band containing key information in relation to dietary change (Table 3, Fig. 4). Sequencing identified b812 as *Collinsella aerofaciens* from the phylum of *Actinobacteria* and b496 as *Clostridium sp.* from the phylum of *Firmicutes*.

**Table 3:** Changes in microbiota due to intervention in all participants; shown are bands with a significant change (0→1: band is absent before and present after intervention; 1→0: vice versa);^ #^ *p*Value of McNemar test.

Band	Intervention	Change in appearance of band		*p* ^#^
		0 → 1	1 → 0	
^1^b300°	Red meat	0	4	0.05
^5^b496*		0	4	0.05
b501		5	0	0.03
^6^b543		0	4	0.05
^8^b601		0	5	0.03
b664		4	0	0.05
^9^b710		0	4	0.05
b935°		5	0	0.03
	Whole grain			
^2^b323		7	1	0.03
^3^b337		6	0	0.01
^4^b378		4	0	0.05
b466		1	7	0.03
b557		1	7	0.03
^7^b594		7	1	0.03
^10^b812*		4	0	0.05
b968		0	4	0.05

* Bands remained significant in multivariate logistic regression model with intervention as dependent variable and all changed bands as independent variables.

°Change in microbiota only in group starting interventions with WG intervention.

Separate analyses on the appearance of bands after the intervention periods revealed that after both intervention periods, more bands were present in a higher number of participants compared to baseline. After the WG and RM intervention 19 and 15 bands, respectively, were present in more than 50% of the participants. After the WG diet, diversity increased by 4 bands per sample, whereas it remained stable after the RM intervention (Table 2).

The interventions did not significantly change SCFA concentrations and calprotectin measured in faecal samples (Table 2).

### 3.3 Factor analysis

The factor analysis ascertained 5 factors with an eigenvalue greater 1. The proportions of explained variance were 31.1%, 30.9%, 19.8%, 12.4% and 12.2% for the factors 1 to 5 with respective eigenvalues of 4.3, 2.9, 1.8, 1.3 and 1.1. The ICCs for DGGE data were between 0 and 1 (mean (SD): 0.39 (0.25)); 52 out of 128 were significant indicating that the 80 observations cannot be treated as completely independent, a fact that has to be considered in the interpretation of the factors and their correlations.

The dietary interventions were significantly related to 3 of these factors; factor 1 to RM and factors 2 and 4 to WG (Table 4). After orthogonal rotation, factor 1 was negatively loaded by all bands that increased after RM intervention and it was positively loaded by bands that decreased after RM intervention. Factor 2 showed similar loading patterns since it was negatively loaded by bands rising after WG and positively loaded by bands declining after WG intervention. Despite the relation between factor 4 and the WG intervention no clear association between the factor and the 16 bands that changed after one of the diets could be detected.

**Table 4:** Intervention effects on factors retained after factor analysis (FA) with bands changed due to an intervention and correlations of corresponding factors with measures of obesity (BMI-body mass index, Waist circ.- waist circumference) and sex.

FA included changed bands (n = 16)			Correlations of factors with			
Factor (Eigenvalue/explained variance)	Intervention effect		Measures of obesity			Sex
	RM	WG	BMI	Weight	Waist circ.	
	*p*-Values		Correlation coefficients			
1 (4.3/31.3%)	0.001	0.29	−0.28*	−0.27*	−0.30*	0.23*
2 (2.9/30.9%)	0.22	<0.0001	−0.22*	−0.13	−0.12	0.06
3 (1.8/19.8%)	0.76	0.22	−0.06	−0.07	−0.07	0.05
4 (1.3/12.4%)	0.50	0.009	0.21*	0.18	0.19	−0.20*
5 (1.1/12.2%)	0.25	0.70	−0.05	−0.03	−0.02	−0.08

* Correlation significant on level *p*<0.05.

Factor 1 was inversely associated with BMI, weight, and waist circumference and positively correlated with sex. Factor 2 was inversely associated with BMI only, and factor 4 was positively correlated with sex and negatively with BMI. None of these associations persisted after adjustment for sex (Table 4).

## Discussion

In this crossover intervention study, we investigated the isocaloric exchange of RM with WG products for a direct influence on gut microbiota composition. For each diet, we identified 8 DGGE bands with in both cases either one bacterium (*Collinsella aerofaciens (Actinobacteria)* for WG) or bacterial group *(Clostridium sp. (Firmicutes)* for RM) carrying the key information on the relation between the respective foods and intestinal microbiota. The study indicated that a diet rich in WG products increased microbial diversity. Further, we showed that the diets altered anthropometric and blood parameters.

The randomised crossover design enabled us to control inter-individual variability appropriately despite the small number of participants and to identify effects across individuals. High adherence of the study participants was secured by detailed instruction, provided food and regular contact and was also indicated by elevated serum levels of creatinine and uric acid after RM intervention, since both substances are components or metabolites of RM. Duration of the interventions was limited to 3 weeks, since study participants can only adhere to such regimens for a short period of time. However, the time was sufficient to observe changes in microbiota composition.

An interesting finding is the higher microbial diversity after the WG intervention which seems to be relevant in the context of other studies that reported a reduced diversity in obese and diseased individuals [19], [20]. Also regarding colorectal cancer risk the microbial diversity seems to be relevant since other studies found the diversity decreased in cancer cases [21].

Agreeing with, but more far-reaching than studies showing effects of fibre supplements on intestinal microbiota [22], we demonstrated direct effects of common foods, i.e. WG bread and RM on gut microbiota composition. To our knowledge, previous food-based interventions in healthy, normal weight individuals have only been performed with fruits and vegetables [23], [24]. We clearly associated the WG intervention with a higher intake of dietary fibre, a substrate for a large proportion of intestinal bacteria. It is known that non-starch polysaccharides, e.g., hemicellulose, promote the growth of commensal gut bacteria [25]. For RM intake, no direct effects on the proliferation of bacterial cells have been established so far, but meat-derived substances, such as nitrite, may also affect microbiota composition [26] and its effects on health [27].

The changed bands, particularly *Clostridium sp.*, belong to predominant bacterial groups in the intestinal microbiota [28], [29]. It is not surprising, however that findings on the health effect of *Clostridium sp.* are inconsistent since the group is large and includes many species. The anaerobic bacterium is already present in the intestine of 30 days old newborns [30]. A study investigating the effects of prebiotics found *Clostridium sp.* decreased after a fibre rich diet, whereas this genus decreased after ingestion of red meat in our study [31]. However, from our data we cannot suggest the exact species from this group that was changed through a RM diet. Since many species from the *Clostridium sp.* are related to pathogenic conditions [32], the reduction of this genus of bacteria may also have beneficial effects. On the other hand studies found a lower abundance of *Clostridia* in colorectal cancer cases [21]. At least, we did not find any adverse results after a 3-week intervention with RM as assessed by our set of anthropometric and biochemical variables. In line with other reports, *C. aerofaciens* changed after the WG intervention. Walker and colleagues found *C. aerofaciens* decreased after a reduced carbohydrate, high protein diet [33]; in our study we could observe the bacterium more often after the WG period with low amounts of protein due to reduced RM intake. This bacterium is more prevalent in faeces of healthy subjects than in Crohn's disease patients [34] and it is inversely associated with irritable bowel syndrome symptoms supposing beneficial effect of this species on gut health [35].

A factor analysis of the 16 changed bands detected inter-correlations between microbiota bands and revealed 5 factors explaining most of the variance within microbiota data. Interestingly, the retained factors were related to measures of obesity and sex. Most of the associations between factors and measures of obesity could be explained by differences between men and women in regard to obesity. Calculated ICCs revealed that DGGE bands are not only correlated across individuals but also within participants. That may lead to an overestimation of the variance explained by the generated factors. It was not possible to clearly separate the respective proportions contributed by intra- and inter- individual covariance for each factor. To address this problem, future studies that apply advanced statistical methods with a larger number of participants are needed.

Although the interventions were designed to be isocaloric, we found a reduction in weight, BMI and body fat mass after the WG intervention indicating that WG products influence energy utilization. Similar effects of WG intake on anthropometry have been observed in cohort studies as well as in previous intervention studies [36], [37]. Since the diets and changes in anthropometry were accompanied by a shift in microbiota, one can hypothesize that the altered microbiota composition is responsible for the energy utilization effect [6]. A reduction in total body fat was also observed in studies with mice harbouring different microbiota [3]. Additionally, it appears that individuals differing in microbiota composition possess different abilities of energy regulation and fat storage resulting in differential risks to gain weight [3], [38]. Underlying mechanisms include distinct macronutrient exploitation from diet and differentially regulated gene expression [39].

On the other hand, our data on the simultaneous changes in microbiota composition and anthropometric parameters should induce a debate about the interpretation of the findings. We claim that diet effects obesity and microbiota and is the underlying factor inducing associations between microbiota composition and obesity. Thus, an association between gut bacteria and obesity may be due to WG intake. This hypothesis is in agreement with previous studies mostly conducted in mice that attributed the capability to play a role in the development of obesity to intestinal microbiota [39]. Further support is provided by cohort studies finding biomarkers of obesity reduced in participants with high WG intake [7]. In the present study, we did not detect any differences in biomarkers such as total cholesterol or serum triglycerides due to the WG intervention, which probably can be attributed to the brevity of 3-week intervention period.

A limitation of the study is the method of intestinal microbiota analysis. The DGGE bands characterise the microbial composition and diversity as well as shifts within the community, but they do not give information on the abundance and concentration of separate bacterial species [40]. We compensated this limitation by sequencing the two bands that carried the key information. A further limitation is the manner of collecting and analysing faecal samples representing microbiota composition from the gut lumen, but not from the entire gut [41].

In conclusion, this study showed that an elevated consumption of RM and WG products, respectively, modifies intestinal microbiota and is associated with a simultaneous change in measures of obesity. The increase of the appearance of *C. aerofaciens* through a fibre rich diet supports the assumption of health promoting effects of WG products. The finding of the study provides guidance to focus on particular microbiota bands (and strains) in observational studies to obtain further evidence on the relation of diet, gut microbiota and health outcomes within large cohorts.

## Supporting Information

Checklist S1 **CONSORT Checklist.**
(DOC)

Protocol S1 **Trial Protocol.**
(DOCX)
