# Supplementary material for: The Influence of Whole Grain Products and Red Meat on Intestinal Microbiota Composition in Normal Weight Adults: A Randomized Crossover Intervention Trial
Source: PLoS One. 2014 Oct 9;9(10):e109606. doi: 10.1371/journal.pone.0109606 (PMC4192132; doi:10.1371/journal.pone.0109606)
Supplement: Protocol S1 — Trial Protocol. (DOCX) [file pone.0109606.s002.docx]

**Study protocol- intervention study**

**Name**: Work package 2 of the TORNADO-project: Individual intervention studies (Adults), www.fp7tornado.eu

**Study centre**: German Institute of Human Nutrition

Department Epidemiology

Arthur-Scheunert-Allee 114-116

14558 Nuthetal

Germany

**Administration**: Prof. Heiner Boeing

Tel.: 033200-88-710

Fax: 033200-88-721

Email: boeing@dife.de

**Partner**: Karolinska Institutet

S Petterson/ J Rafter

Department of Microbiology, Tumor and Cell Biology

Stockholm, Sweden

Umeå University

Olle Hernell MD, PhD

Department of Clinical Sciences

Umeå, Sweden

Queen Mary and Westfield College

Thomas T. MacDonald, PhD

Centre for Infectious disease

London, UK

Institute of Food Research

Dr. Arjan Narbad

Norwich Research Park

Norwich, UK

The study will be conducted according to the ethical principles of the declaration of Helsinki in its latest version (52^nd^ WMA General Assembly, Edinburgh, Scotland, October 2000).

**Financing:**

Seventh Framework Programme (FP7)- project TORNADO

Content

Research project

Background

Aim

Participants

Recruitment

Exclusion criteria

Study

Strategy

Examinations/ Sampling

Interventions

Processing of samples

Time schedule

Ethics

Risks and inconvenience

Insurance coverage

Information of participants regarding examination results

Data protection

Expense allowance

Supplement

Supplement 1 Study information for participant

Supplement 2 Informed consent

Supplement 3 List of cooperating institutes and laboratories

Research project

Background

Life style factors, such as physical activity, smoking and dietary habits influence the presence of chronic diseases, such as cardiovascular diseases, inflammatory bowel diseases and type 2 diabetes. Diet for example can influence the composition of the intestinal microbiota by intake of probiotics and scientific studies admit the assumption of a relation between the intestinal microbiota composition and the development of chronic diseases.

Among humans being overweight- one of the risk factors for chronic diseases- a changed ratio of the two most frequent bacterial groups, Firmicutes and Bacteroidetes, was determined in the gut.

Intestinal bacteria enable energy extraction from actually indigestible dietary compounds for the human body by different means.

A changed intestinal microbiota composition is accompanied by changes in the energy metabolism, the fat storage and in mechanisms of the immune system.

All these components also may contribute to the development of chronic diseases.

Until today it is unclear if also the daily diet with its individual preferences can influence the intestinal microbiota composition.

Potentially a diet rich in fibre encourages the growth of specific bacteria, whereas a diet rich in red meat encourages the growth of other bacteria.

The present study aims to investigate the mentioned associations more detailed among healthy adults.

Aim

Investigation of the influence of a special diet on the physical constitution, the intestinal microbiota and the metabolism in healthy adults.

**Participants**

Healthy adults aged 20 to 60 years

Recruitment

20 Adults (20- 60 years) without chronic or acute diseases;

10 men and 10 women, three to five participants in each of the following age categories:

- 20-30 years
- 31-40 years
- 41-50 years
- 51-60 years

Exclusion criteria

- - Chronic diseases (diabetes, cardiovascular diseases, …)
  - Antibiotic treatment within 3 month before study start
  - Gastrointestinal diseases (e.g. intestinal bowel disease)
  - gastrointestinal disorders (e.g. colon irritabile)
  - gastrointestinal surgeries
  - celiac disease
  - cancer

Additionally the intake of probiotics within the last two weeks before study start should be mentioned with the product’s name.

Study

Strategy

Every participant will be informed regarding the study, the interventions and the possibly related risks in a detailed talk with the study doctor at the beginning of the study.

Further, this talk provides the opportunity to clarify all questions of the participant regarding the study. Subsequent to the talk the participant will confirm his or her participation written.

First week of study dietary habits of participants will be assessed by a questionnaire. Additionally the physical baseline condition will be assessed. Therefore body weight, body height, waist and hip circumference, and circumference of upper arm and leg will be determined after one week during a second visit in the study center. These data serve the calculation of body mass index (BMI) and the general characterization of participant’s physical constitution. A blood sample (maximum 65ml) will be drawn, partly investigated regarding metabolic and hormonal biomarkers within the following 24 hours and partly splitted into its fractions (serum, plasma, buffy coat, cellular fractions) and stored at -80°C for later analyses. Further, urine is sampled and stored for further analyses. Additionally a saliva sample is taken, which will be investigated regarding its components and the microbial composition.

Finally, the participant is requested to provide a fecal sample, which will be investigated regarding the composition of intestinal microbiota and the presence of characteristic substances in the gut. The participant samples this fecal sample at home, all necessary material will be provided. Fecal sampling should take place within two days after the visit in the study center. The Fecal sample will be collected by an institute’s employee within one week.

The one-week run-in period is followed by two three-week intervention periods, which will be separated by a three-week wash-out period.

The mentioned examinations and samplings will be repeated after each intervention and after the wash-out period during an appointment in the study center

Overall, the participant will be in the study center at five appointments, of which four will be examination appointments.

Examinations

 anthropometry:

- body weight measurement
- body height measurement
- blood pressure measurement
- waist cirdumference
- hip circumference
- upper arm circumference
- leg circumference
- measurement of skinfold thickness
- measurement of breast depth and width

 blood sampling

 urine sampling

 saliva sampling

 fecal sampling

Intervention

Following the first examination every participant follows either the following diet:

 very little red meat and simultaneously high amounts (at least 35g/day) of fiber (low red meat and high meal bread (fiber) 🡪lmhf)

or

 high amounts (200g/day) red meat and simultaneously little amounts of fiber (high red meat and low meal bread (fiber)🡪 hmlf)

Precise amounts of foods during the interventions are given in table 1.

These amounts of special foods will be integrated in the habitual diet; they will not be consumed additionally.

Regarding other food groups (beverages, frutis/vegetables), dairies, alcohol …) the participant follows his/her habitual manner.

Diets will be isocaloric, i.e. calory intake will not differ between the two interventions.

Table 1: Amounts of foods, which will be consumed during the interventions

| Food | | Recommendation for a balanced diet | | Diet during hmlf | | Diet during lmhf | |
| --- | --- | --- | --- | --- | --- | --- | --- |
| fiber (from whole grain bread, -pasta, -rice, -cereal) | | about 30g/day | | Less than 25g/day | | **At least 35g/day** | |
| Red meat (mammalian meat, no poultry, no fish) | | Less than 80g/day | | **200g/day** | | Less than 30g/day | |

These diet will be adhered to for three weeks (intervention period 1). Following these three weeks there will be another examination in the study center, which will be the same compared to the first one.

It is followed by a three-weeks wash-out period, during which the participant will follow his or her habitual diet. Also after these three weeks the above mentioned examinations will follow.

During the next three weeks the intervention period 2 will follow, during which the participant will follow the opposed diet compared to intervention period 1. If he or she followed during the first period the pattern lmhf than it will be hmlf or conversely. After this three weeks there will be the last examination, again consisting of anthropometric measurements, sampling of blood, urine and saliva.

Processing of samples

Blood samples

By the blood sampling and the analysis of the blood different biomarkers can be determined and by this an association between diet and physical constitution can be investigated.

The sampling will be made in the study center by a study doctor.

One part of the blood will be analyzed within 24h (e.g. blood fat, CRP; HbA1c, GOT/GPT). The other part will be processed within two hours and stored at -80°C in its fractions (serum, plasma, buffy coat, cellular fractions). These fractions will be used for later extraction of DNA and PBMC (peripheral blood mononuclear cells), by which additional biomarkers will be analyzed.

For these investigations a blood sample of max. 65ml will be drawn from every participant at each examination appointment.

Urine samples

At the appointment day in the study center every participant will be asked to provide an urine sample (about 50ml). This will be stored in a monovette system until processing. A part will be processed contemporary, another part will be stored at -80°C for later analyses.

Saliva samples

The participant will be asked to collect 1 to 2 ml saliva in a sterile small tube. It will be mixed with special substances to avoid protein degradation and cell decomposition during storage.

Subsequently the sample will be stored deep-frozen until further analysis. Similar to the fecal sample there will be an investigation of the microbiota in saliva additionally the sample will be investigated regarding compounds, such as glucose and amylase.

Fecal samples

The sampling will be done by the participant at home. Therefor the institute will provide a fecal backup aid, two fecal tubes and packaging material. The participant will sample by using the fecal backup aid and the spoon in the tubes, the filled sampling tube will be packed in a packaging tube, a plastic bag and a plastic box. The sample will be stored at -20°C (freezer compartment of the fridge or freezer) until it will be collected by an institue’s employee within 7 days. In the institute the sample will be stored at -80°C until processing A part will be sent to Norwich, UK for intestinal microbiota analyses (16SrRNA analysis by a “gutflora chip”). Another part will stay at the institute for analyses of fatty acids (short chain fatty acids and branched chain fatty acids), ammonium and fecal water (comet assay)

Time schedule

For every participant the study will last ten weeks from the first appointment tot he final examination.

Figure 1: Time schedule of the intervention study

Time schedule for the complete study

Study start: November 2010

Planned completion of data acquisition: March 2011

| **Week** | **1** | **2** | **3** | **4** | **5** | **6** | **7** | **8** | **9** | **10** | **11** | **12** | **13** |
| --- | --- | --- | --- | --- | --- | --- | --- | --- | --- | --- | --- | --- | --- |
| **activity** | **run in** | | |  |  |  |  |  |  |  |  |  |  |
|  |  | **Interventions period 1** | | | | |  |  |  |  |  |  |  |
|  |  |  |  |  | **washout** | | | | |  |  |  |  |
|  |  |  |  |  |  |  |  | **Interventions period 2** | | | | |  |
| **Unter-suchung** |  | **Baseline** | | | **Intervention 1** | | | **washout** | | | **Intervention 2** | | |
|  |  |  |  |  |  |  |  |  |  |  |  |  |  |

**Ethic**

Risiks and inconveniences

It is assumed that there will be no harms or disadvantages for participants due to study participation.

Some examinations (measurement of skinfold thickness, fecal sampling) may will be perceived inconvenient by some participants.

The blood drawing will be done by a study doctor. Possibly hematoma will occure. These are in most cases minimal and reoccur autonomous within a few days. Risks and side effects such as increased bleedings of infections on the puncture side are utterly seldom.

The participant should not be disturbed in his habitual daily routine by the intervention. The daily getting and eating of the stated amounts of whole grain products and red meat should be integrated in the daily routine without problems.

Insurance coverage

An insurance for a „clinical examination that is not liable to a compulsory insurance” will be concluded.

No path insurance for participant’s way to the study center will be concluded.

Participants will be informed regarding the insurance conditions at the start of the study.

Information of participants regarding examination results

Participants have the possibility of a personal nutrition advice talk, which will provide dietary recommendations considering latest research results.

Information resulting from the analyses of fecal and blood samples are only useful in a scientific context, therefor the results will be not communicated. Also on an individual disease risk participants will get no information.

Finally, all participants will get information on the findings regarding the intake of red meat and whole grain products.

Data protection

Data will be saved according to the „Brandenburger Datenschutzgesetz” and to the agreements with the “Datenschutzbeauftragten des Landes Brandenburg”. All Information will be stored and analysed without a direct link to identifiying data in pseudomynised manner

Personal data will be stored coded on a special saved computer and autarkic server, respectively. Only for possible queries of selected study employees a manufacture of the name reference can be necessary.

Expense allowance

Participants get a general expense allowance of 200€ after completion of all examinations. Foods will be provided by the institute therefore no further costs arise due to study participation

**STUDY INFORMATION PROVIDED TO THE PARTICIPANTS**


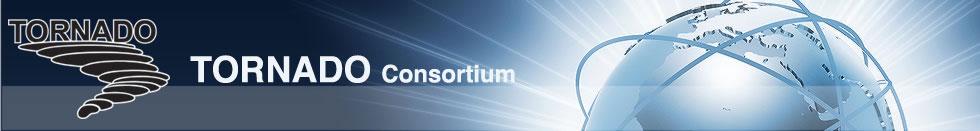


**INFORMATION**

Individuelle Interventionsstudie zur Untersuchung des Zusammenhangs zwischen einer speziellen Ernährung und der Zusammensetzung der Darmmikrobiota sowie der Körperkonstitution und des –metabolismus

Studienleitung:

Deutsches Institut für Ernährungsforschung (DIfE)

Abteilung Epidemiologie

Prof. Heiner Boeing

Nuthetal, Deutschland

Kooperationspartner:


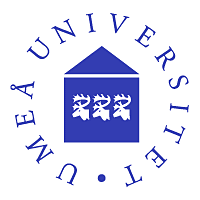
Umeå University

Olle Hernell MD, PhD

Department of Clinical Sciences

^ Umeå, Sweden


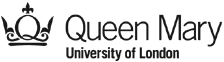
Queen Mary and Westfield College

Thomas T. MacDonald, PhD

Centre for Infectious disease

London, UK


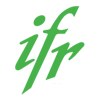
Institute of Food Research

Dr. Arjan Narbad

Norwich Research Park

Norwich, UK

**Ziele**

Diese Studie ist Teilprojekt des europäischen Programms TORNADO (Molecular Targets Open for Regulation by the gut flora – New Avenues for improved Diet to Optimize European health), welches in Kooperation mit dem Karolinska Institut in Stockholm und 20 weiteren europäischen Instituten und Lebensmittelherstellern durchgeführt wird. Das Projekt untersucht die Auswirkungen der Ernährung auf die Entwicklung der Darmmikrobiota und des Immunsystem über die gesamte Lebensspanne hinweg. Diese Effekte werden auf metabolischer Ebene und auch auf anatomischer Ebene ermittelt.

**Teilnehmer**

Teilnehmer sind gesunde Frauen und Männer ohne chronische und akute Erkrankungen zwischen 20 und 60 Jahren.

Ausschlusskriterien:

- - chronische Erkrankungen (Diabetes, Herz-Kreislauf-Erkrankungen,…)
  - Antibiotikatherapie innerhalb der letzten 3 Monate vor Beginn der Studie
  - gastrointestinale Erkrankungen (z.B. entzündliche Darmerkrankung)
  - gastrointestinale Störungen (z.B. Reizdarm)
  - gastrointestinale Operationen
  - Sprue (celiac disease)
  - Krebserkrankungen

Zusätzlich sollte die Einnahme von probiotischen Lebensmitteln innerhalb der letzten 2 Wochen vor der Studie unter der Nennung des Produktnamens angegeben werden.

**Interventionen**

Während der Studie sollen speziell die Auswirkungen einer ballaststoffreichen Ernährung (vor allem mit Vollkornprodukten) bzw. eines hohen Anteils von rotem Fleisch (Rind, Schwein etc.) in der täglichen Ernährung auf den menschlichen Körper untersucht werden. Dafür ernähren sich die Teilnehmer jeweils für 3 Wochen auf die eine oder die andere Weise.

Vor Beginn der Intervention werden während einer einwöchigen run in-Periode die gewohnte Ernährungsweise und der körperliche Ausgangszustand des Probanden ermittelt (Baseline). In den folgenden 3 Wochen ernährt sich der Teilnehmer von viel Vollkornprodukten und sehr wenig rotem Fleisch (low red meat and high meal bread (fiber) 🡪 **lmhf**) oder umgekehrt von viel rotem Fleisch und wenig Vollkorn (high red meat and low meal bread (fiber) 🡪 **hmlf**). Die genauen Mengenangaben sind in Tabelle 1 beschrieben.

Nach diesem Zeitraum werden erneut die Daten und Maße des Probanden ermittelt und Proben genommen. An die erste Interventionsphase schließt sich ein 3-wöchiger Zeitraum an, in welchem sich der Teilnehmer wie gewohnt ernährt (washout). Dies dient zur „Auswaschung“ der Effekte der speziellen Ernährung in den vorangegangenen Wochen.

Es folgen weitere 3 Wochen, in welchen sich der Proband genau entgegengesetzt zur ersten Interventionsphase ernährt. Hat er also zunächst viel rotes Fleisch und wenig Vollkorn (hmlf) gegessen, dann folgt jetzt die Phase, in der er wenig rotes Fleisch und viel Vollkorn (lmhf) zu sich nimmt (Interventionsphase 2).

Tabelle 1: Vorgaben zur Ernährung während der zwei Interventionsphasen lmhf (low red meat and high meal bread (fiber)) bzw. hmlf (high red meat and low meal bread (fiber)); *Empfehlung der Deutschen Gesellschaft für Ernährung

| **Lebensmittel** | **Empfehlung für eine aus-gewogene Ernährung*** | **Ernährung während lmhf** | **Ernährung während hmlf** |
| --- | --- | --- | --- |
| Ballaststoffe aus  Vollkornprodukten  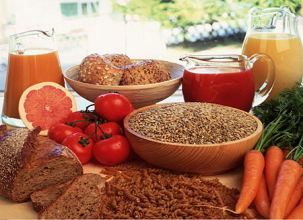 | etwa 30g/Tag | **mind. 35g/Tag**  (200-300g Vollkorn-produkte/  Tag | unter 25g/Tag |
| Rotes Fleisch (Säugetierfleisch,  kein Geflügel,  kein Fisch)  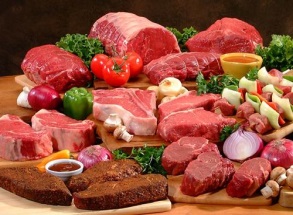 | unter 80g/Tag | unter 30g/Tag | **200g/Tag** |

**Welche Untersuchungen werden durchgeführt?**

Jeder Proband stellt sich 5 Mal im Studienzentrum des DIfE (Lageskizze s. Seite 8) vor. Der erste Termin (visit 1) dient zur genauen Information des Teilnehmers und beinhaltet ein Aufklärungs- und ein Beratungsgespräch durch qualifiziertes Personal.

An allen weiteren Terminen werden Untersuchungen vorgenommen und Proben entnommen. Außerdem besteht auch während dieser Termine immer die Möglichkeiten Fragen des Teilnehmers zu klären. Der zweite Termin findet nach der run in Phase und vor Beginn der ersten Intervention statt (visit 2). Die nächsten Untersuchungen (visit 3) werden nach der ersten Interventionsphase durchgeführt. Der nächste Termin findet nach der 3-wöchigen washout Phase statt (visit 4) und abschließend ist die Untersuchung nach der zweiten Interventionsphase nach nochmals 3 Wochen (visit 5).

Bei allen Terminen außer dem ersten werden bei jedem Probanden anthropometrische Messungen (Körpergröße, Gewicht…) durchgeführt. Zusätzlich werden eine Blutprobe (max. 65ml), eine Speichelprobe und eine Urinprobe abgenommen. Außerdem bekommt der Proband Material mit nach Hause, um eine Stuhlprobe zu nehmen. Diese wird zunächst beim Teilnehmer eingefroren und anschließend von einem Mitarbeiter des Instituts abgeholt.

Tabelle 2: Art und Inhalte der Untersuchungen während der Studie

| **Untersuchung** | **Inhalte** |
| --- | --- |
| Anthropometrie | Körpergewicht, Körperhöhe, Taillenumfang, Hüftumfang, Arm- und Beinumfang, Hautfaltendicke, Blutdruck, Brustkorbtiefe, Brustkorbbreite |
| Probenentnahme | Blutprobe, Urinprobe, Speichelprobe, Stuhlprobe |

**Zeitplan der Studie**

Die gesamte Studie erstreckt sich über einen Zeitraum von insgesamt 10 Wochen, in welchen der Proband zu 5 Terminen ins DIfE kommt. Wobei an 4 Tagen Untersuchungen durchgeführt werden. Je Termin sollte der Teilnehmer einen Zeitaufwand von etwa 60min einplanen.

Abbildung 1: Zeitplan der Studie

**Der Weg ins Studienzentrum**

Das Studienzentrum befindet sich auf dem Gelände des Deutschen Instituts für Ernährungsforschung (Gelände E) in der Arthur-Scheunert-Allee 114-116 in Potsdam-Rehbrücke.

Lage des Studienzentrums im DIfE


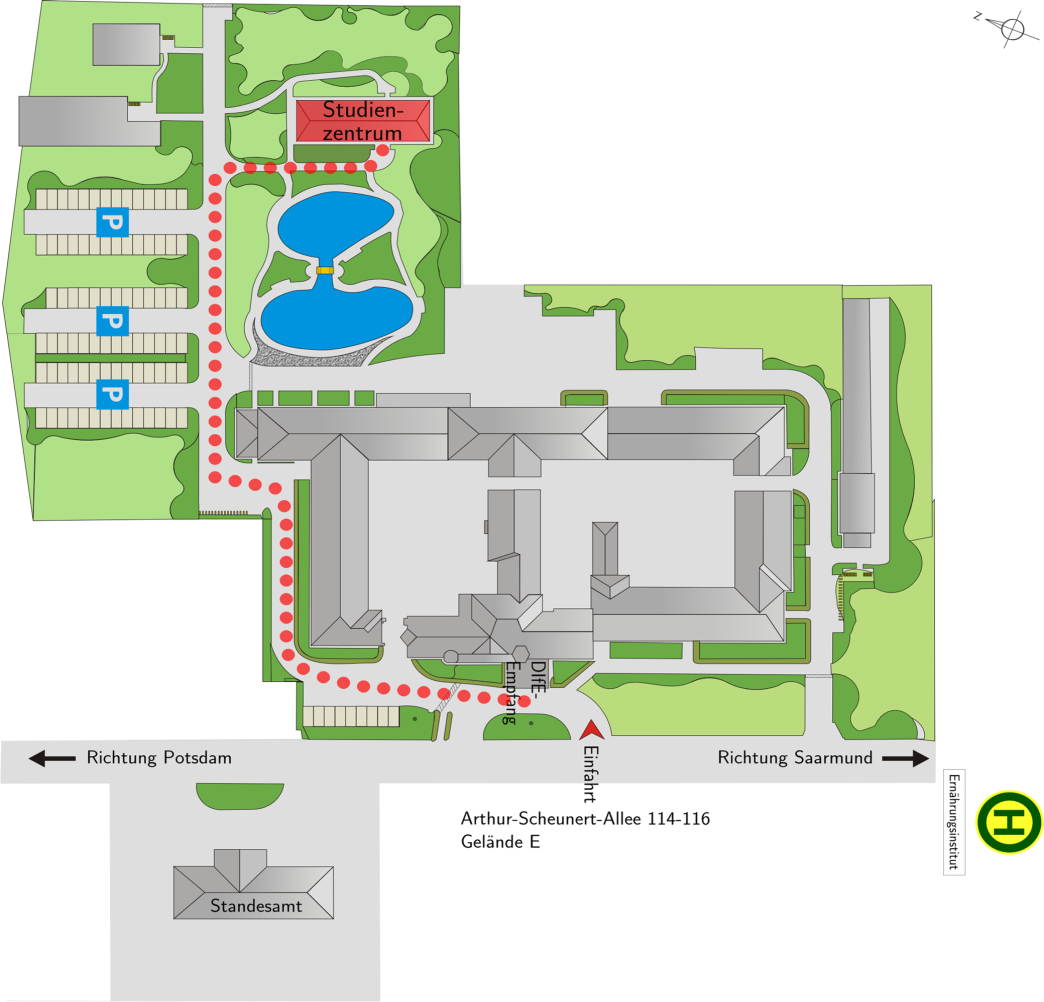


Abbildung 2: Lage des Studienzentrums auf dem Gelände E des DIfE

Anfahrt

- Mit dem PKW

Auf dem Gelände des DIfE stehen Parkplätze zur Verfügung, die während des Besuches im Studienzentrum genutzt werden können.

- Öffentliche Verkehrsmittel

Bahn: RE7 oder MR33 von Wannsee aus bis Potsdam-Rehbrücke

Tram: Linie 91 und 93 bis Endhaltestelle Bahnhof Rehbrücke von Potsdam Hbf aus

vom Bahnhof Potsdam-Rehbrücke sind es 1,3km Fußweg auf der Arthur-Scheunert-Allee in Richtung Saarmund

Bus: Linie 693 bis Haltestelle Verdistraße, 1km Fußweg auf der Arthur-Scheunert-Allee in Richtung Saarmund, Linie 691 bis Haltestelle Ernährungsinstitut

Ansprechpartner für Fragen oder Probleme:

Jana Förster

-Abteilung Epidemiologie-

jana.foerster@dife.de; 033200-88-731

**INFORMED CONSENT**


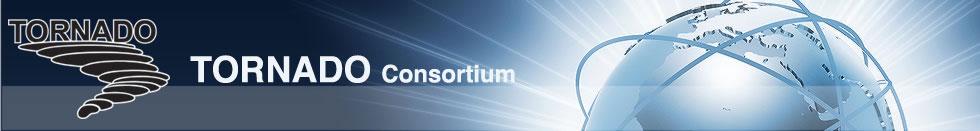
Interventionsstudie

im Rahmen des TORNADO Projekts

am Deutschen Institut für Ernährungsforschung- Potsdam

**Einverständniserklärung**

Teilnehmerdaten

Anrede: Herr/Frau

Name: ______________ Teilnehmer-Nummer: _________

Vorname: ______________

geb. am: ___.___.______

Ich habe die Informationen zu oben genannter Studie erhalten, gelesen und verstanden.

Ich erkläre mich bereit:

- für jeweils 3 Wochen an den in der Information beschriebenen Interventionen teilzunehmen
- 5 Termine im Studienzentrum wahrzunehmen und dabei an 4 Terminen folgende Untersuchungen durchführen zu lassen:
  - Anthropometrie, Blutdruck, Puls
  - Blutentnahme (65ml)
  - Urin-, Speichel und Stuhlprobe abgeben

Ich bin damit einverstanden, dass meine während der Studie gewonnenen Daten in pseudonymisierter Form gespeichert und im Rahmen wissenschaftlicher Kooperationen mit Forschern im In- und Ausland zwecks Datenanalysen, die der Erreichung des beschriebenen Studienziels dienen, weiter gegeben werden dürfen.

Ich habe verstanden, dass Informationen mit persönlichem Bezug auf meine Person nicht an Dritte weitergegeben werden. Veröffentlichungen der Studienergebnisse in wissenschaftlichen Zeitschriften beinhalten lediglich Berichte von Personengruppen, ohne Rückschlüsse auf einzelne Personen zuzulassen.

Ich bin mit der unbefristeten Lagerung und Nutzung der von mir gewonnenen Proben (Blut, Stuhl, Urin, Speichel) und den daraus abgeleiteten Bestandteilen (Zellen, Stoffwechselwege beschreibende Substanzen, Erbinformation) einverstanden.

Ich habe verstanden, dass die Untersuchungen ausschließlich der Beantwortung wissenschaftlicher Fragestellungen und daher nicht im medizinischen Sinne dem Ausschluss von Erkrankungen oder der Stellung einer Diagnose dienen.

Mir ist bewusst, dass die Teilnahme an der Studie freiwillig ist und ich jederzeit ohne Angabe von Gründen zurücktreten kann.

Nuthetal, ______________________

___________________________________/________________________________________

Teilnehmer(in) Druckschrift Teilnehmer(in) Unterschrift

___________________________________________

List of the cooperating institutes and laboratories

German Institute of Human Nutrition

Department Epidemiology

Prof. Heiner Boeing

Nuthetal, Deutschland

Karolinska Institut

Department of Biosciences and Nutrition

The TORNADO Project

Sweden

www.fp7tornado.eu

Umeå University

Olle Hernell MD, PhD

Department of Clinical Sciences

Umeå, Sweden

Queen Mary and Westfield College

Thomas T. MacDonald, PhD

Centre for Infectious disease

London, UK

Institute of Food Research

Dr. Arjan Narbad

Norwich Research Park

Norwich, UK
